# Biopolymer-Based Hydrogel Incorporated with Naproxen Sodium and Lidocaine Hydrochloride for Controlled Drug Delivery

**DOI:** 10.3390/polym16101353

**Published:** 2024-05-10

**Authors:** Dorota Wójcik-Pastuszka, Karolina Stawicka, Witold Musiał

**Affiliations:** Department of Physical Chemistry and Biophysics, Faculty of Pharmacy, Wroclaw Medical University, ul. Borowska 211A, 55-556 Wrocław, Poland; dorota.wojcik-pastuszka@umw.edu.pl (D.W.-P.); karolinastawicka98@gmail.com (K.S.)

**Keywords:** biopolymer, polysaccharide, biocompatible polymer, differential scanning calorimetry, drug release, naproxen sodium

## Abstract

Sodium hyaluronate (HA) is a natural polysaccharide. This biopolymer occurs in many tissues of living organisms. The regenerating, nourishing, and moisturizing properties as well as the rheological properties of HA enable its application in the pharmaceutical industry as a carrier of medicinal substances. The aim of this work was to assess the release of naproxen sodium (Nap) in the presence of lidocaine hydrochloride (Lid) from the biopolymer-based hydrogels and to determine the respective kinetic parameters of this process. The possible interaction between the HA polysaccharide carrier and the selected drugs was also investigated. Three hydrogels containing Nap and Lid with different concentrations of the biopolymer were prepared. The release of Nap was studied by employing USP apparatus 5. The infrared study and differential scanning calorimetry analysis of physical mixtures and dried formulations were performed. The highest amount of Nap was released from the formulation with the lowest concentration of the biopolymer. The most representative kinetic model that described the dissolution of Nap was obtained through the Korsmeyer–Peppas equation. The release rate constants were in the range of 1.0 ± 0.1 × 10^−2^ min^−n^–1.7 ± 0.1 × 10^−2^ min^−n^. Lid did not influence the dissolution of Nap from the formulations tested; however, in the desiccated samples of assessed formulations, the interaction between the polysaccharide and both drugs was observed.

## 1. Introduction

Polymers can be used as gelling agents in hydrogels, which are semi-solid forms of drugs. Hydrogels are cross-linked polymers that have the ability to bind large amounts of water [1]. Thang et al. [2] widely studied the polymer-based hydrogels proposed as drug delivery systems, especially for prolonged drug application. The three-dimensional crosslinking structure of hydrogels allows for the penetration of large amounts of water into the interior, as well as medicinal substances, genes, and other molecules, which can be delivered to the tissues and released in the targeted place. One of the advantages of using the local application of hydrogels (for example, on the skin in wound therapy) is that it allows for the reduction of side effects. Mahmood et al. [3] focused, in their work, on researching the biopolymer-based hydrogels, and mainly the polysaccharides and polypeptides as well as nucleic acids. Several groups of hydrogels were distinguished, depending on the stimulus to which they are sensitive and their applications in biomedicine. The response of the hydrogel to changes in the environmental temperature, pH, charge, and light or under the influence of enzymes determines its ability to control drug release. The possibility of combining different hydrogels sensitive to various stimuli was also found. The unique properties of biopolymers can be employed to obtain sophisticated carriers for drug delivery, the regeneration of damaged structures, and ligament treatment.

One of the biopolymers used to obtain hydrogels is hyaluronic acid, which is often found in the form of a sodium salt (HA). HA occurs in living organisms. It is a component of the extracellular matrix of tissues, and it was also found in the fluids of vertebrates. In the human body, it is present in the synovial fluid, the vitreous body, and the skin. It has protective, moisturizing, and nourishing functions, and it participates in cell regeneration [4,5,6]. The natural origin and physicochemical properties of HA ensure its biocompatibility, its low toxicity, and the absence of allergic reactions [7].

**Figure 1:**
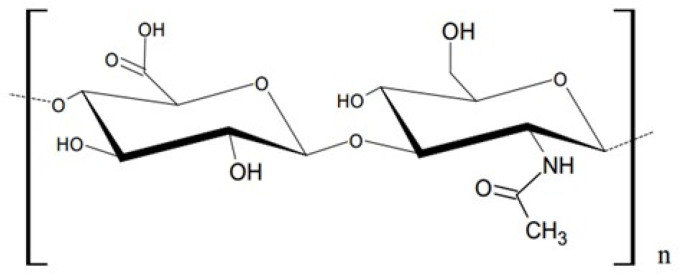
The HA disaccharide structure.

HA is a natural polysaccharide consisting of disaccharide units consisting of alternately arranged D-glucuronic acid (GlcA) and N-acetyl-D-glucosamine molecules (GlcNAc). Between these subunits, glycosidic β (1–3) and β (1–4) bonds exist [8]. The structure of this biopolymer is presented in Figure 1.

The molecular weight of a single disaccharide is approximately 400 Da. However, the molecular weight of HA occurring in physiological conditions ranges from 4000 to 10,000 kDa. This biopolymer has anti-inflammatory properties and protects against the adverse effects of free radicals. HA with low molecular weight increases inflammatory processes, apoptosis, and angiogenesis. It is produced as a result of HA degradation under, e.g., the influence of hyaluronidase enzymes [9,10].

The high-molecular-weight HA has the ability to bind large amounts of water. Combined with it, it forms a sticky, clear gel. The solubility of HA in water is a result of the presence of four hydroxyl groups in a single disaccharide unit [10]. These properties of HA enable its application in cosmetics, e.g., as a dermal filler, and also in pharmaceutical technology as a drug carrier. However, the internal, endogenous, high-molecular HA in human tissues is not stable due to the activity of hyaluronidases. They break long HA chains, which are consequently removed from the organism. To enhance the stability of the hydrogels composed of high-molecular-weight exogenous HA applied to human tissues, cross-linking agents are additionally introduced to the preparation. They bind together the long polymeric chains, which results in a more stable carrier [9].

**Figure 2:**
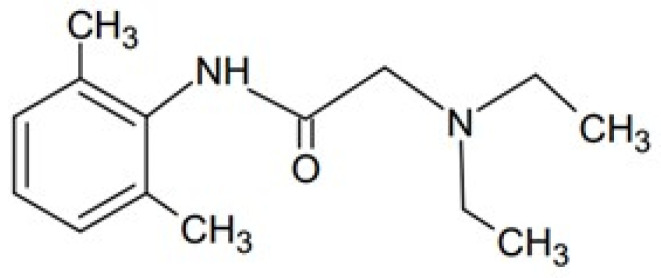
The structure of Lid.

Lidocaine (Lid) is a local anesthetic widely applied in ambulatory and emergency healthcare. It consists of a hydrophobic aromatic ring and a lipophobic amino group that are connected by an amide or ester linkage. The structure is presented in Figure 2.

Lid has in its structure the amide group that enables it to form hydrogen bonds [11]. It was found that the incorporation of Lid into the porous hydrogel decreases the pore size. This is connected to the filling of the network space with the drug molecules interacting with the polymer through the formation of hydrogen bonds, which, as a consequence, results in the compression of the hydrogel space. The porous structure is very important in biomedical applications of hydrogels, especially in tissue regeneration [12].

Lid occurs very often in the ionized form as lidocaine hydrochloride salt. It is a weak basic compound with a pKa value of 7.9, resulting in ca. 25% of Lid in a salt form at the physiological pH of 7.4. In pharmacy, it is used in the form of patches, aerosols, injection solutions, ointments, or gels. The therapeutic dose of Lid in gels, patches, or ointments ranges from 2% to 5% [13,14,15].

**Figure 3:**
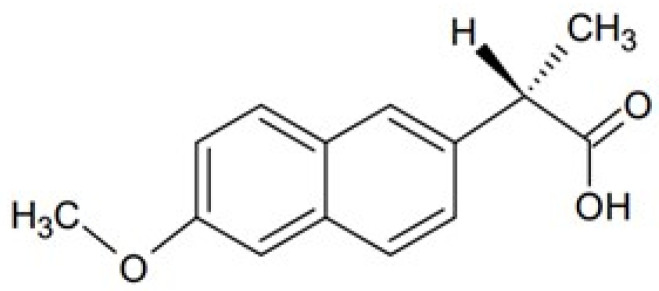
The structure of Nap.

Naproxen (Nap) is usually used in pharmaceutical technology in the form of a sodium salt. It is classified as a non-steroidal anti-inflammatory drug, and it exhibits analgesic, antipyretic, and anti-inflammatory effects. It consists of two condensed benzene rings, which enable the prolonged activity of the drug [16]. The structure of Nap is presented in Figure 3. The Nap molecule contains a chiral center, so it can exist in the form of a pair of enantiomers. Both Nap structures have similar physical characteristics, but available studies indicate greater activity of the S(+) enantiomer in the inhibition of thromboxane compared to the R(−) enantiomer. For this reason, the pharmaceutical market employs the pure S(+) enantiomer [17].

Nap is an acidic compound with a pKa of 4.15. Nap is marketed in the form of tablets, capsules, gels, and suppositories. The maximum total daily dose of Nap is 1500 mg [18,19].

Žid et al. [20] studied the influence of surface functionalization on Nap’s release from mesoporous silica. It was found that at pH = 7.4, Nap was released faster from the surface containing a negatively charged carboxylate ion than from the system functionalized with phenyl groups. Under these conditions, Nap exists in the form of an anion; therefore, it is transported faster to the acceptor fluid due to the repulsion of the acidic anion. However, the presence of phenyl groups hydrophobizes the silica surface. Such a carrier in the interaction with the Nap ring reduces the solubility of the drug. The same results were obtained by Halamova et al. [21]. The researchers postulated that amino groups on the surface increase its hydrophobicity. The Nap molecules are mostly in the interior rather than on the surface, which, as a result, reduces its release.

A therapy composed of both drugs was proposed by Ferhad et al. [22]. However, Nap was delivered orally, and Lid was in the hydrogel dosage form. The matrix of this formulation was based on synthetic polymers. In the present study, we proposed a new formulation based on the natural polymer, HA, with both drugs incorporated into hydrophilic HA gel. This work was a continuation of previous studies concerning the influence of the HA on the dissolution patterns of anionic and cationic drug introduced into the hydrogel. The anionic drug was sodium naproxen (Nap) and the cationic substance was lidocaine hydrochloride (Lid) [23]. In the present work, we studied the release of Nap from HA-based matrixes in the presence of Lid. The purpose of this work was to test the Lid’s influence on the dissolution of Nap from HA-based hydrogels. It was interesting to calculate the kinetic parameters of Nap release from formulations containing Lid and to compare the parameters with the values obtained in the previous study, where Nap was released from hydrogels without Lid [23]. Moreover, the interactions between the ingredients of the composition were also studied. The idea was to prepare a formulation containing Nap and Lid at the same concentrations as in the preparations used by the patients. The concentration of Nap in the hydrogels present on the market is 10%, whereas the concentration of Lid in the marketed preparations is 2%, according to the available data of compositions registered, inter alia, by the FDA or the EMA.

## 2. Materials and Methods

HA (sodium hyaluronate) was delivered from Esent (Szczecin, Poland), and Lid (lidocaine hydrochloride) was obtained from Sigma-Aldrich (Steinhelm, Germany). Nap (naproxen sodium) was a free sample from Hasco Lek (Wrocław, Poland). Sodium hydroxide and potassium dihydrogen phosphate were bought from Chempur (Piekary Śląskie, Poland). The phosphate buffer pH = 7.4 was drawn up in accordance with the European Pharmacopoeia [24]. The cellulose membranes came from Carl Roth (Karlsruhe, Germany).

### 2.1. Hydrogel Preparation

The required amount of Nap was weighed and dissolved in water. The required quantity of Lid was also weighed and added to water. Both solutions were added to the proper amount of HA, thus obtaining compositions F1, F2, and F3 with polymer concentrations of 1.5, 2.0, and 2.5%, respectively. The obtained hydrogels were mixed using a homogenizer (Unidrive X 1000D, Cat, Staufen, Germany) with a rotation speed of 16,000 rpm for 10 min until the homogenous formulations were formed. The compositions of hydrogels are shown in Table 1.

The preparations were stored for 24 h at a temperature of 6 °C to remove air bubbles. The concentration of Lid in gels used as local anesthetics approved by the FDA is 2% [25]. However, for adults, they should be given no more than 600 mg of Lid within 12 h [26]. The Nap concentration present in market hydrogels is 10% [27,28]. In this work, the same doses of drugs were incorporated into the prepared biopolymer compositions.

### 2.2. Viscosity Study

The viscosity study was carried out using the rotational viscometer (DV2T, Brookfield, Middleboro, MA, USA). The hydrogels were heated in a water bath until the temperature reached 37 °C. The measurement was performed for 1 min using the dedicated spindle at a rotation speed of 200 rpm. Each test was repeated 5 times to calculate the mean value of the dynamic viscosity (dynamic viscosity coefficient) and the standard deviation (SD).

### 2.3. Release Study

Nap’s dissolution from preparations was tested by employing apparatus 5, with the paddle over the disk (ERWEKA, DT 126 Light, Heusenstamm, Germany), according to Ph. Eur. 10.5 [29]. The proper amount of the hydrogel was introduced into six extraction cells. The drug was released through cellulose membranes into the acceptor fluid with a volume of 1 L. The acceptor medium was phosphate buffer pH = 7.4 prepared according to Ph. Eur. 10.5 [29]. The release study was performed at a temperature of 37 °C with a paddle rotation speed of 50 rpm. The samples of 3 mL were taken out at defined time intervals, and the dissolution vessels were replenished with the fresh fluid. The measurements were carried out for 490 min. The amount of Nap released was determined spectrophotometrically at a wavelength of 330 nm. At this wavelength, Lid is invisible, and HA does not give a maximum over the entire UV-Vis range.

### 2.4. Difference Factor and Similarity Factor

The evaluation between the dissolution curves was performed in accordance with FDA recommendations [30] for determining the difference coefficient f_1_ and the similarity coefficient f_2_ using Equations (1) and (2), presented below:

(1) $$f_{1}=\frac{\sum_{t=1}^{n}\left(R_{t}-T_{t}\right)}{\sum_{t=1}^{n}R_{t}}\times 100$$

(2) $$f_{2}=50\times log\left\{{\left[1+\frac{\sum_{t=1}^{n}{\left(R_{t}-T_{t}\right)}^{2}}{n}\right]}^{-0.5}\times 100\right\}$$

where n is the number of time points, R_t_ is the dissolution value of the reference batch at time t, and T_t_ is the dissolution value of the test batch at time t.

The profiles are considered similar if the f_1_ value is in the range of 0 to 15 and the f_2_ value is between 50 and 100.

### 2.5. Kinetic Study

The kinetic analysis was carried out using zero-, first-, and second-order kinetics as well as Higuchi and Korsmeyer–Peppas dependences [31,32,33,34]. The equations were included in our previous work [23,35,36], and they are as follows:

(3) $$\mathrm{Zero}\text{-}\mathrm{order}\;(Z\text{-}O)\;\;m_{t}=m_{b}+k_{0}t$$

(4) $$\mathrm{First}\text{-}\mathrm{order}\;(F\text{-}O)\;\;\ln\left(m_{0}-m_{t}\right)=\ln\left(m_{0}\right)-k_{1}t$$

(5) $$\mathrm{Sec}\mathrm{ond}\text{-}\mathrm{order}\;(S\text{-}O)\;\;\frac{1}{\left(m_{0}-m_{t}\right)}=\frac{1}{m_{0}}-k_{2}t$$

(6) $$\mathrm{Higuchi}\;(H)\;\;\;m_{t}=k_{H}t^{0.5}$$

(7) $$\mathrm{Korsmeyer}\text{–}\mathrm{Peppas}\;(K\text{-}P)\;\log\left(\frac{m_{t}}{m_{\infty }}\right)=\log k_{K-P}+n\log t$$

where m_t_ is the amount of the drug released in time t; m_b_ is the amount of the drug in the solution before the release (usually, it is 0); k_0_ is the zero-order release rate constant; m_0_ is the amount of the drug in the formulation before the dissolution; k_1_ is the first-order release rate constant; k_2_ is the second-order release rate constant; k_H_ is the Higuchi rate constant; m_∞_ is the amount of the drug released after infinitive time; k_K-P_ is the Korsmeyer–Peppas rate constant; n is the parameter indicative of the drug release mechanism; and m_r_ is the amount of the drug remaining in the formulation in time t.

The zero-order kinetics model assumes that the drug dissolution rate is constant and that it does not depend on the concentration of the released substance. At subsequent time intervals, the same amount of the substance is released. It is most commonly used to describe drug release from transdermal patches, matrix tablets with poorly soluble drugs, and hydrophilic gels [37]. The first-order kinetics describes the process in which the release rate of a medicinal substance is proportional to the drug’s concentration. The amount of the drug released to the acceptor medium is proportional to the amount of the drug remaining in the formulation, decreasing in time unit. It is applied to characterize the drug transport from porous matrices incorporated with highly water-soluble drugs [38]. In the second-order model, the release rate is proportional to the square of the drug concentration. In the Higuchi model, it is assumed that the drug transport process follows Fick’s law of diffusion. The amount of the released drug is directly proportional to the square root of time. This means that the substance is released faster at the beginning, and the process slows down in the later stages. This is the model most commonly used to describe the release of an active substance from a modified drug release form, such as, for example, from transdermal patches [39]. The Korsmeyer–Peppas model approximates the release of the drug from the polymer matrix and, due to the presence of the n parameter in the equation, allows for determining the mechanism of drug transport from the carrier to the acceptor fluid. The release rate involves the structural and geometric characteristics of the dosage form [40].

Based on these mathematical relationships, the kinetic parameters, such as the release rate constants, the half release time, and the Korsmeyer–Peppas model’s n factors, were determined.

### 2.6. Statistical Analysis

The mean values of the presented parameters were calculated as an arithmetic average with a standard deviation (SD). The kinetic analysis was based on the least squares method at a confidence level of 95%. The comparison of the respective release profiles of Nap from the studied hydrogels was performed using Student’s *t*-test at a significance level of *p* = 0.05.

### 2.7. FTIR Measurements

The FTIR (Fourier Transform Infrared) spectra were recorded using a spectrometer with ATR mode (Nicolet iS50, Thermo Scientific, Waltham, MA, USA). The measurements were carried out at a resolution of 16 cm^−1^ in the wavenumber range of 4000 to 400 cm^−1^. The samples were dried at a temperature of 6 °C and crushed in a mortar before analysis. In total, 32 scans of each sample were recorded, with a speed of 65 scans per 1 min.

### 2.8. DSC Measurements

The DSC (differential scanning calorimetry) study was performed using a calorimeter (214 Polyma, Netzsch, Wittelsbacherstraße, Germany). Hydrogels for the DSC investigation were prepared similarly to those for the FTIR analysis. Then, 3–5 mg samples were placed in aluminum crucibles, covered with a lid, pressed, and placed in the calorimeter. The measurements were carried out in a temperature range of −10 to 300 °C under nitrogen’s presence. The flow rate of the gas was established at 25 mL/min.

## 3. Results and Discussion

### 3.1. Viscosity Study

The measured viscosity of the formulations F1–F3 containing Nap and Lid, in comparison to the viscosity of the corresponding Lid-free hydrogels assessed in previous work [23], is listed in Table 2. It was found that the viscosity of the preparations F1–F3 was lower than the corresponding viscosity of hydrogels with Nap only. The introduction of Lid to the formulations F1–F3 reduced the viscosity of the tested preparations.

The reduction of F1–F3’s viscosity may also indicate interaction between Nap and Lid drugs or an additional interaction between Lid and the macromolecule. The tendency towards increasing viscosity with increased biopolymer concentration was observed both in the case of formulations containing Lid and without Lid. This is consistent with the literature data [41], in which the viscosity increased with the addition of the polymer to the hydrogel. A decrease in viscosity was observed at low shear rates, which increased at higher viscosities. The increased number of macromolecules in the formulation increases the contact between polymer chains and thus increases intermolecular interactions. This is due to the larger amount of hydrogen bonds, mainly with hydroxyl groups. It was found that hydrated molecules of a solute, e.g., a drug dissolved in water, affect the velocity of the liquid, causing an increase in viscosity. Additionally, the increased number of molecules in a system results in the increase of the viscosity [42].

### 3.2. Release Study

**Figure 4:**
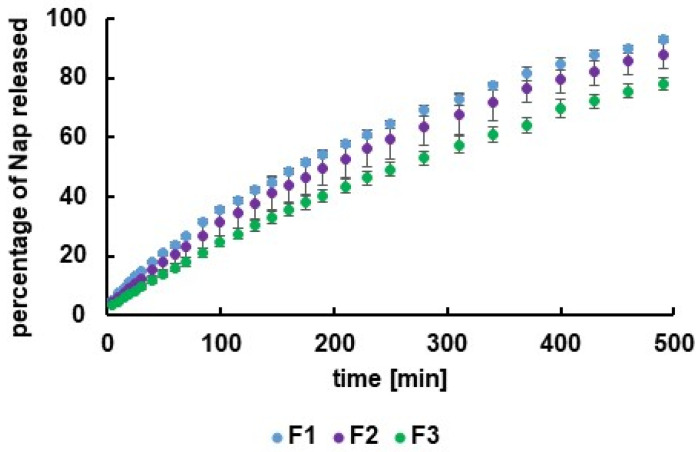
The Nap dissolution profiles obtained from hydrogels F1–F3.

The Nap release plots for hydrogels F1–F3 are presented in Figure 4.

It was observed that the highest amount of the drug after 490 min of the study was released from the F1 hydrogel, with the lowest amount of the biopolymer. The lowest concentration of Nap in the acceptor fluid after 490 min of the test was found in the case of the dissolution of the active substance from preparation F3, with the highest concentration of the polymer and, therefore, the highest viscosity. An enlargement in the concentration of the polymer matrix, and thus growth in its viscosity, prolongs the transport of the drug from the hydrogel to the external environment. This result may indicate that the increase in the carrier concentration in the formulation and, therefore, the increase in its viscosity may allow for obtaining a hydrogel with controlled drug release. The drug may be transported to the acceptor fluid slower and in lower amounts, and a sustained release effect can be achieved.

### 3.3. Release Curves Assesment

The dissolution profiles were assessed by determining the difference factor f_1_ and the similarity factor f_2_. The obtained values of these coefficients are collected in Table 3.

According to FDA recommendations [30], values of f_1_ below 15 ensure sameness between the curves, meaning that the dissolution profiles of Nap from formulation F1 and F2 were the same, although the differences between the release curves of Nap from F1 and F3 and from F2 and F3 were revealed. In the case of f_2_, a value below 50 indicates differences. The analysis of f_2_ confirmed the similarity between the Nap release profiles from F1 and F2 and the differences between the drug dissolution curves from F1 and F3. However, a discrepancy in the case of the assessment of the Nap release curves from F2 and F3 was noticed. The value of the f_1_ factor indicated a difference, but the value of the f_2_ coefficient indicated similarity. The statistical analysis using Student’s *t*-test showed that there were no significant differences between all of the compared profiles. The differences between the polymer concentrations in the tested formulations F1–F3 were small, and this may explain the lack of differences or the difficulty in noticing differences between the compared Nap release profiles. The dependence between the drug release and the polymer concentration in the carrier was studied by Das et al. [43]. It was revealed that increased concentration of the polymer in the hydrogel reduced the Nap dissolution. It was explained that as the hydrogel concentration increased, the matrix changed its structure from porous to dense, and, simultaneously, the swelling rate decreased and hindered the drug’s transport into the acceptor fluid.

### 3.4. Kinetic Analysis

The obtained release data were fitted to zero-, first-, and second-order equations as well as Higuchi and Korsmeyer–Peppas models by employing the least squares method [38]. The results of this kinetic analysis are summarized in Table 4.

**Figure 5:**
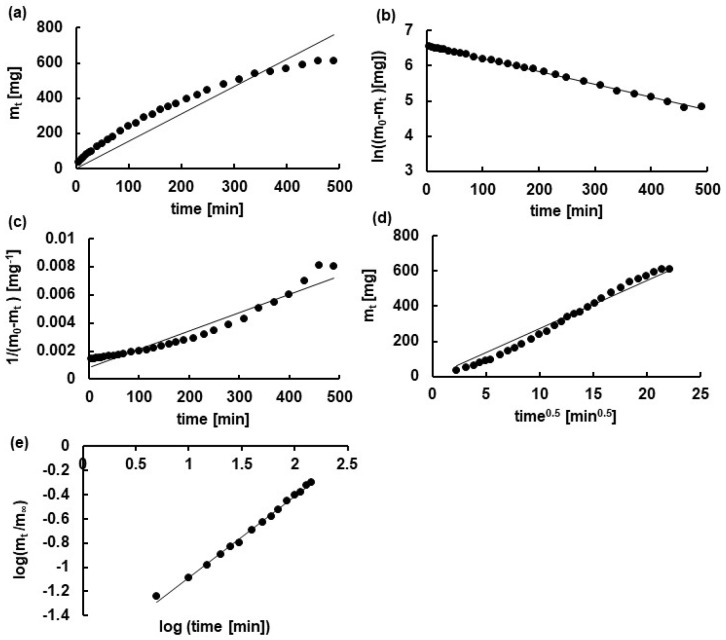
The fitting of the experimental points for F1 to the theoretical curves of the models: (**a**) zero−order (**b**); first−order (**c**); second−order; (**d**) Higuchi; (**e**) Korsmeyer−Peppas.

The example of fitting the experimental data to the theoretical equations is presented in Figure 5. It should be noticed that the Korsmeyer–Peppas equations were the models that most properly described the observed dissolution process. The values of the correlation coefficients were very high, and they were 0.99 ± 0.01 for each formulation. The values of the release rate constants, calculated according to the Korsmeyer–Peppas equation, were (1.7 ± 0.1) × 10^−2^ min^−n^, (1.5 ± 0.1) × 10^−2^ min^−n^, and (1.0 ± 0.1) × 10^−2^ min^−n^ for the formulations F1, F2, and F3, respectively, thus indicating that the drug was transported the fastest from formulation F1. The amount of the carrier polymer in hydrogel F1 was the lowest, as well as the viscosity. Nap was released at the lowest rate from hydrogel F3, which indicated the highest amount of HA and the highest viscosity of the hydrogel.

We noticed that the first-order model is also suitable for characterizing the drug transportation from the polymer matrix to the acceptor fluid with the value of R^2^ from 0.98 ± 0.01 to 0.99 ± 0.01. Also, in this case, Nap was released at the highest rate from the preparation with the lowest concentration of the polymer and the lowest viscosity. It is interesting that in our previous work [23], the release of Nap from Lid-free hydrogels was also most sufficiently described using the Korsmeyer–Peppas equation with the R^2^ value of 0.99 ± 0.01. Additionally, the values of the release rates calculated using the Korsmeyer–Peppas model for Nap release from Lid-free hydrogels were in the range of (1.78 ± 0.18) × 10^−2^ min^−n^ to (1.42 ± 0.16) × 10^−2^ min^−n^, and they were very close to the release rate constants obtained in the present work. Moreover, the study of Nap release from Lid-free hydrogels revealed that the release rate constant was the highest when the drug was released from the formulation with the lowest carrier concentration and the lowest hydrogel viscosity [23], and this was consistent with the results obtained in this work. The kinetics of the Nap release from different hydrogels or transdermal systems was also analyzed by employing zero- and first-order equations as well as Higuchi and Korsmeyer–Peppas models [44,45]. It was found that the best model, depending on the formulation, was Korsmeyer–Peppas or zero-order, although the Higuchi model and the first-order equations also had very high coefficient R^2^ values.

The values of the constant n in the Korsmeyer–Peppas model obtained in this study in all cases were in the range of 0.67 ± 0.01–0.74 ± 0.02, supporting the idea that Nap was released from the polymer carrier to the acceptor medium via an anomalous transport. The interpretation of the value of the parameter n by Costa and Lobo [38] characterizes values of n between 0.5 and 1.0 as an indication of the drug’s release via an anomalous transport. The results obtained in the present study correlate very well with the data from previous studies of Nap’s release from Lid-free hydrogels, where the values of the n constant were in the range of 0.69 ± 0.03 to 0.72 ± 0.04, also indicating the anomalous transport [23]. This kind of transport may be explained by the hindered movement of Nap molecules between densely packed polysaccharide chains, which are additionally connected by cross-linking.

These results also corresponded well with the data obtained from the study of the Nap permeation from Carbopol 980 gels through the skin. In this case, the process was analyzed using the Korsmeyer–Peppas equation, and the calculated value of the parameter n was in the range of 0.5 < n < 1.0, indicating the anomalous transport [46]. The study of the kinetics of drug release from hydrogels indicated that the release of the dissolved substance is composed of two processes: the polymer swelling resulting from the penetration of solvent molecules into the interior, and the drug’s transport from the matrix to the liquid. It was found that such a process usually follows the anomalous transport triggered by the molecular relaxation of the polymer, which is connected to the drug’s diffusion [47].

It can be summarized that the incorporation of a second drug, i.e., Lid, into the biopolymer-based hydrogel does not affect the release of Nap. The absence of the effect may be explained by the interaction between Lid and HA instead of the interaction between Lid and Nap. According to the study by Mrestani et al. [48], Lid dissociates into the chloride anion and the Lid cation, whereas sodium hyaluronate dissociates into the sodium cation and the HA anion. Therefore, from an electrostatic point of view, an interaction between the positive Lid ion and the negatively charged HA is possible. Additionally, the abovementioned team revealed the bond formation between Lid and glucuronic acid GluA, which is one of HA’s components. Thus, according to our experimental results, and Mrestani evaluations of a parallel system, binding of Lid to HA may shorten the Lid’s influence on the Nap release.

### 3.5. FTIR Study

**Figure 6:**
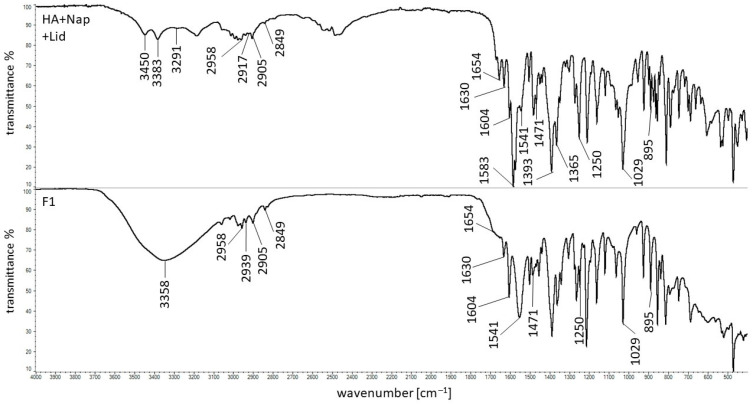
The FTIR spectra of the physical mixture of HA, Nap, Lid, and the formulation F1 (composition in Table 1).

The FTIR spectra of the physical mixture composed of Nap, Lid, and HA together with the preparation F1 are shown in Figure 6. The spectra of pure ingredients, such as Nap, Lid, and HA, and the preparation consisting of HA with residual water, are presented in previous work [23,36], where they may be compared to the present data. The characteristic bands of HA at 3291, 2917, 2849, and 1604 cm^−1^ were found on the record of the physical mixture, illustrated in Figure 6. It is worth noting that the weak bands at 1407 and 1375 cm^−1^ were not visible because they were overlapped by strong signals from Nap at 1393 cm^−1^ and 1365 cm^−1^. The sharp peak at 1029 cm^−1^ may be assigned to the presence of HA as well as Nap. The signal of HA at 895 cm^−1^ was also noticed on the spectrum of the physical mixture in Figure 6. The peak at 1743 cm^−1^ belonging to the deprotonated group –COOH of the polymer was invisible, indicating the presence of the form without a proton –COO^−^. All characteristic bands of Lid at 3450, 3383, 1654, 1541, and 1471 cm^−1^ were observed on the spectrum of the physical mixture. The maxima at 2958, 2905, 1630, 1583, and 1250 cm^−1^ indicating the presence of Nap in the physical mixture were also noticed.

The analysis of the spectrum of formulation F1 presented in Figure 6 revealed that the signals of Lid at 3450 and 3383 cm^−1^ disappeared, and the peak at 1654 cm^−1^ was very weak. Similar results were obtained in the previous study [23]. The absence of these maxima on the spectrum of the preparation consisting of Lid, HA, and water indicated the interaction between HA and Lid. In the present work, the interaction between these components was confirmed. Additionally, the weak interaction between Lid and HA was also found by Mrestani et al. [48].

The maxima at 2958, 2905, 1630, and 1250 cm^−1^ related to the presence of Nap were observed on the spectrum of formulation F1. However, the band at 1583 cm^−1^ belonging to the stretching of the COO^−^ group of Nap was not noticed. This was consistent with the analysis obtained in the previous study [23]. The sharp signal at 1583 cm^−1^ on the FTIR spectrum of the formulation consisting of HA, Nap, and water was also not observed, suggesting bond formation between Nap and HA. This was consistent with the observation reported by Larrañeta et al. [49]. In that study, the researchers revealed the ester bond formation between the hydroxyl group belonging to the HA molecule and the carboxyl group coming from the Gantrez S97 polymer.

Considering the distinctive maxima of HA on the F1 record, it was found that the wide signal at 3358 cm^−1^ was observed, while the signal at 3281 cm^−1^ was not present. The maximum at 2939 cm^−1^ was noticed instead of the peak at 2917 cm^−1^. This may be explained by the overlapping of the HA bands with water signals in this region. Moreover, the discrepancies may also confirm the postulated interaction between HA and Lid and the interaction between HA and Nap. The bands of HA at 1407 and 1375 cm^−1^ were not present, similarly to the spectrum of the physical mixture. The signals of HA at 2849, 1604, 1029, and 895 cm^−1^ were found. The spectra of formulations F2 and F3 composed of the same ingredients were analogous to F1.

The FTIR analysis confirmed the interaction between HA and Lid as well as between the polymer and Nap. The observed changes on the spectrum of the formulation compared to the spectrum of the physical mixture may also be the result of interactions between drugs. However, studies on the kinetics of Nap release from the formulation with and without Lid did not show any differences.

### 3.6. DSC Analysis

**Figure 7:**
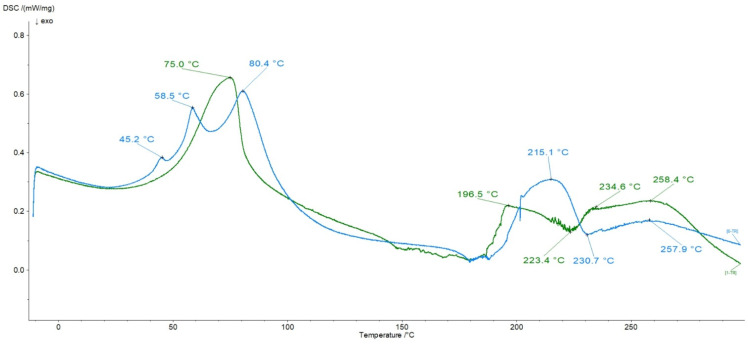
The thermograms of the physical mixture of HA, Nap, and Lid and formulation F3—composition in Table 1.

The thermograms of pure ingredients were published in our previous work [23,36]. The DSC curves of the assessed physical mixture of HA, Nap, and Lid and the thermogram of preparation F3 are shown in Figure 7.

On the plot of the physical mixture, the endotherm of Lid at temperatures of 75.0 and 196.5 °C was observed. The peak at 258.4 °C belonging to Lid was also found, although it can also be related to the endotherm of Nap that occurred on the DSC curve of pure Nap at 257.3 °C [23]. The very weak signal of Nap at 62.2 °C associated with water loss was invisible on the presented thermogram. The HA endotherms corresponding to the water loss at 69.0 and 124.2 °C were not noticed. They were covered with a stronger Lid signal at the temperature of 75.0 °C. The weak exotherms of the biopolymer degradation at 223.4 and 234.6 °C were present. However, it should be mentioned that the thermogram of the physical blend of the components differed from the thermogram of formulation F3, which can be noticed in Figure 7. On the heating curve of formulation F3, three endotherms below 100 °C were present. The endotherm at 80.4 °C was attributed to the slightly shifted Lid signal. The peak at 257.9 °C related to Lid was also observed, although it can be related to the endotherm of Nap as well. The signal of Lid at 195.5 °C disappeared, and a new endotherm at 215.1 °C emerged. A similar observation was reported in the previous study, as the heating curve of the preparation consisting of HA, Lid, and water indicated an interaction between HA and Lid, which was identified in peaks’ alternations observed between 195 and 220 °C [23]. The new endotherm at 215.1 °C may therefore also be explained by the interaction between HA and Lid. The sharp maximum corresponding to the melting point of Nap was present on the thermogram of the pure compound at 257.3 °C [23]. On the F3 formulation’s DSC curve, in Figure 7, the weak maximum at 257.9 °C was also noticed. It could be overlapping with the signal of Lid. The very weak exotherm that could belong to HA was found at 230.7 °C, although the second one at 246.7 °C disappeared. There is an interesting appearance of three signals at 45.2, 58.5, and 80.4 °C on the thermogram of formulation F3. On the thermogram of the physical mixture of these components, only one endotherm at 75.0 °C was found. The discrepancies may suggest the interaction between Nap and HA, which was postulated in our previous study [23]. Larrañeta et al. [49] proposed the bond formation between the carboxyl group of Nap and the hydroxyl group of HA. This interaction was also proposed in the FTIR analysis of the present work. The interaction between Lid and Nap was not observed in DSC studies. This may be the result of bond formation of Lid and Nap with the polymeric carrier instead of direct interaction between them.

## 4. Conclusions

This study revealed that the presence of Lid in the HA hydrogels did not influence Nap release. The drug was transported the fastest from hydrogel with the lowest polymer amount, which may be connected to the lower viscosity of the preparation. First-order kinetics, Korsmeyer–Peppas, and Hixon–Crowell models described the best Nap dissolution from the evaluated HA hydrogels. It was found that the drug was transported from the biopolymer matrix to the acceptor liquid through an anomalous transport. The interactions between HA and Nap as well as between the HA biopolymeric carrier and Lid were confirmed.

## Figures and Tables

**Table 1:** The compositions of the prepared formulations.

Formulation	F1	F2	F3
HA [g]	3.0	4.0	5.0
Water [g]	173.0	172.0	171.0
L [g]	4.0	4.0	4.0
N [g]	20.0	20.0	20.0

**Table 2:** The viscosity of the preparations incorporated with Nap and Lid (F1–F3) evaluated in this work and the viscosity of the reference hydrogels free of Lid (F1r–F3r) presented in the previous work [23].

Formulation	F1	F2	F3
Viscosity [cP]	1905.2 ± 58.6	4308.0 ± 95.8	8946.0 ± 417.4
Reference formulation *	F1r	F2r	F3r
Viscosity [cP]	2850.1 ± 42.8	5888.0 ± 143.1	13,860.9 ± 638.5

* from [17].

**Table 3:** The values of the difference factors f_1_ and the similarity factors f_2_.

	f1		f2	
Formulation	F2	F3	F2	F3
F1	8.49	31.15	68.54	46.19
F2	―	20.01	―	55.30

**Table 4:** The kinetic parameters of Nap’s release from formulations F1–F3.

Kinetic Model	Kinetic Parameters	F1	F2	F3
Z-O	k_0_ [mg × min^−1^]	1.5 ± 0.1	1.4 ± 0.1	1.4 ± 0.1
	t_0.5_ [min]	219.5 ± 15.5	237.1 ± 14.2	277.0 ± 12.9
	R^2^	0.87 ± 0.01	0.87 ± 0.02	0.95 ± 0.01
F-O	k_1_ ×10^3^ [min^−1^]	4.8 ± 0.3	4.1 ± 0.2	2.9 ± 0.1
	t_0.5_ [min]	144.1 ± 8.2	172.5 ± 7.5	235.9 ± 8.2
	R^2^	0.98 ± 0.01	0.99 ± 0.01	0.99 ± 0.01
S-O	k_2_ × 10^5^ [mg^−1^ × min^−1^]	2.7 ± 0.6	2.0 ± 0.4	0.8 ± 0.09
	t_0.5_ [min]	55.2 ± 11.5	87.2 ± 13.7	161.1 ± 18.6
	R^2^	0.77 ± 0.04	0.84 ± 0.05	0.92 ± 0.01
H	k_H_ [mg × min^−1/2^]	31.5 ± 0.7	24.9 ± 1.1	24.9 ± 1.4
	t_0.5_ [min]	114.2 ± 5.2	186.4 ± 16.6	257.6 ± 28.2
	R^2^	0.97 ± 0.01	0.95 ± 0.01	0.94 ± 0.01
K-P	k_K-P_ × 10^2^ [min^−n^]	1.7 ± 0.1	1.5 ± 0.1	1.0 ± 0.1
	t_0.5_ [min]	160.0 ± 13.8	164.8 ± 19.5	189.5 ± 20.1
	R^2^	0.99 ± 0.01	0.99 ± 0.01	0.99 ± 0.01
	n	0.67 ± 0.01	0.69 ± 0.02	0.74 ± 0.02
best fit		K-P,	K-P, F-O	K-P, F-O

Z-O—zero-order model; F-O—first order model; S-O—second-order model; H—Higuchi model; K-P—Korsmeyer–Peppas model; k_0_—the zero-order release rate constant; k_1_—the first-order release rate constant; k_2_—the second-order release rate constant; k_H_—the Higuchi rate constant; k_K-P_—the Korsmeyer–Peppas rate constant; n—the parameter describing the drug release manner; t_0.5_—half release time; R^2^—the correlation coefficient.

## Data Availability

Data are contained within the article.
